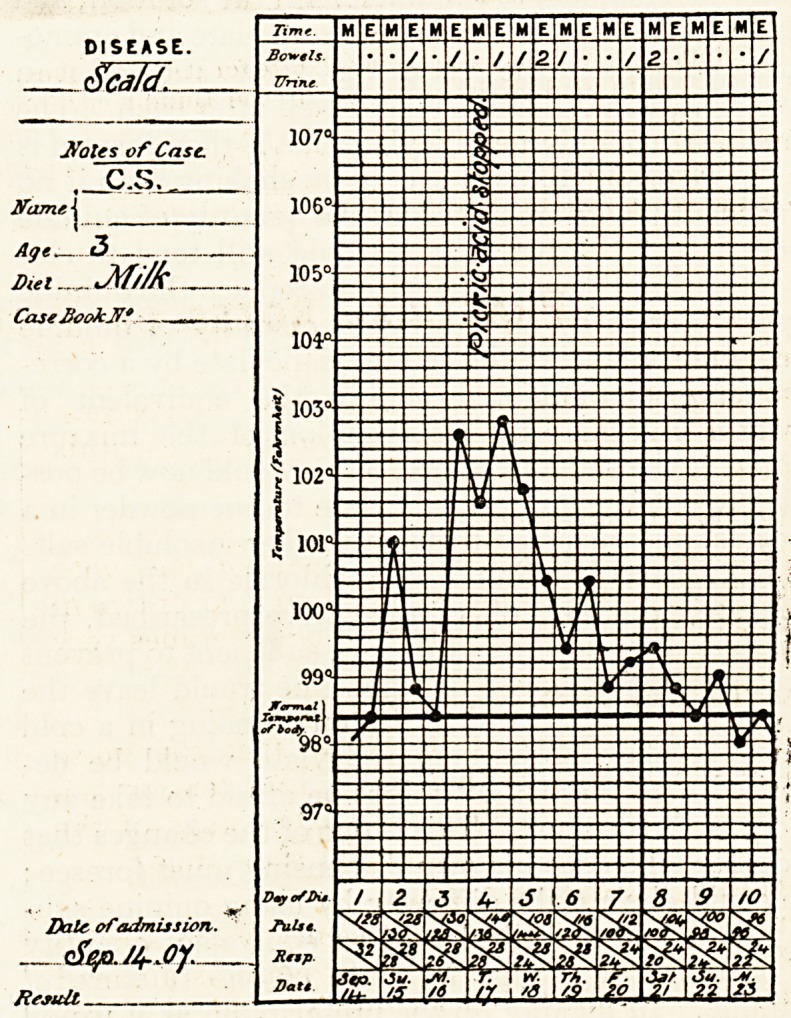# Picric Acid Poisoning

**Published:** 1907-11-30

**Authors:** A. G. L. Reade, J. W. Scott Macfie

**Affiliations:** House Physician and late Senior House Surgeon, The Radcliffe Infirmary, Oxford; House Physician, The Radcliffe Infirmary, Oxford.


### Resident Medical Officers' Department.

[The Editor accepts no responsibility for any opinions expressed in these columns, which are freely open to all resident medical officers. Discussion and contributions are invited, and, if the latter are accepted, they will be paid for.]

### PICRIC ACID POISONING.

By A. G. L. READE, M.R.C.S.Eng., L.R.C.P. Lond., House Physician and late Senior House Surgeon, The Radcliffe Infirmary, Oxford; and J. W. SCOTT MACFIE, B.A.,Cantab., M.B., Ch.B. Edin.; House Physician, The Radcliffe Infirmary, Oxford.

In a previous communication we discussed the "Prognosis in Cases of Burns and Scalds." It may not be inappropriate, therefore, to point out a danger accompanying a very favourite method of treatment, especially as we have had under our observation recently a case admirably illustrating the condition referred to.

Picric acid has proved a most valuable dressing in many cases of burns and scalds, and since it was recommended by Mr. D'Arcy Power, in an article contributed to the *British Medical Journal* for September 12, 1896, has been in constant use in such cases. It has been especially recommended in cases in which the true skin has not been completely destroyed, the vesicles being first punctured and emptied, and picric acid solution being then applied on pieces of lint. But one danger of this treatment is not, perhaps, sufficiently insisted on, and that is the possible supervention of picric acid poisoning. It is a case of this kind that we venture to put on record in the present paper, in the hope that our experience may be of interest to others who are in the habit of using picric acid as a dressing for burns and scalds.

*Case.*—The case is that of a small boy, three years of age, who, whilst playing, scrambled on to the boards covering a cauldron of boiling water. Suddenly one of the planks gave way, and the boy's legs slipped down between the two adjoining pieces of the covering. In this position he was found by his mother, who rescued him, and brought him without delay to the Infirmary.

On admission he was found to have severe scalds on both ankles and feet. Large bullae had already formed, and the skin was partially destroyed. It was thought advisable to keep him under observation for awhile, so he was admitted to one of the wards, and was treated in the first instance with boric ointment. The same night the child's temperature rose, and he became somewhat collapsed, but by next morning he seemed quite comfortable again. The dressing was then changed to picric acid. He had altogether five picric acid dressings, and seemed to be doing well until the afternoon of the third day, when [illegible] observed that a scarlatiniform rash had appeared on his thighs. This spread very rapidly over his body and chest, but did not involve his face. His tongue was heavily furred, and its margin was bright red. He complained of a sore throat and a slight headache; his temperature rose to 102.6, but he had no gastric disturbance and no vomiting. His urine was charged with urates and of a deep orange colour, but it contained no albumen.

So suspicious did the case appear that, in addition to stopping the picric acid dressings, it was thought advisable to isolate the case at once, lest it should prove to be one of scarlet fever.

Meanwhile, the urine was examined more particularly to ascertain if picric acid were present in it. It was accordingly found to give a positive reaction with basic lead acetate and ammonio-cupric sulphate.

On the next day the rash on the body was already beginning to fade away. The boy's temperature was falling, and his tongue was clean and red. He had now some rash out on his face, and his conjunctivæ were tinged with yellow. There was no longer any doubt as to the diagnosis. And from this time until he was finally discharged he made an uninterrupted recovery. In the accompanying temperature chart it will be observed how rapidly the temperature subsided after the picric acid dressings were replaced by boric acid ointment.

Picric acid poisoning is certainly neither a fatal nor a frequent form of poisoning, and only a few cases seem to have been recorded of its occurrence in consequence of the treatment of burns and scalds by this substance. But on account of the unpleasant nature of the symptoms—for severe pain in the stomach, repeated vomiting, and diarrhœa are usually present—and the undoubted resemblance to the exanthems, we have thought it wise to emphasise the necessity for keeping in mind this possible sequel to the employment of picric dressings.

## Figures and Tables

**Figure f1:**